# Mr. Davies, in Answer to Mr. Ring

**Published:** 1807-12

**Authors:** H. Davies

**Affiliations:** Piccadilly


### *To the Editors of the Medical and Physical Journal.*

"Omni tulit punctum, qui miscuit utile, dulce." HOR.

GENTLEMEN,

THE design of the "Remarks," which appeared in a former Number of the Med. and Phys. Journal, was to inculcate the sentiment of our celebrated Latin poet, which I have adopted for my present motto, as it applies to controversial subjects; also to reconcile the views of Practitioners to an alternate mode of treating the same cases under the circumstances of constitutional variety, and to ameliorate, in some degree, the asperity which is too often manifest in Disputants, whenever a collision of sentiment prevails.

My principal attention, when I mentioned "new Theories in Medicine," was to the late discovered mode of treating Burns and Scalds, together with gouty Inflammation; which has been so widely opposite by different practitioners; but as, by repeated experiments, indubitable instances of success have attended either method, owing, perhaps, to the different temperaments of the respective patients; does it not afford a sufficient argument of its kind, why the question in point should in a great measure be at rest?

Together with these Remarks, I added an observation or two on the Vaccine Contest. Here I found, afterwards, I had trodden upon tender ground, and excited a neighbouring practitioner, a zealous champion in this important cause, and to whose indefatigable labours it is much indebted, to make some unfriendly comments on my Paper, to which I will now take the liberty to reply in order.

I have no objection whatever to the improvement Mr. Ring has made in the well-known adage respecting the dead. But allowing what is said of them to be the "*verum,*" instead of the "*bonum,*" if it consists only in recapitulating their errors and prejudices, and holding up their memory to obloquy and contempt, when the individuals themselves are beyond the reach of self-defence; it reflects, in my opinion, but little credit upon the living. I think it would discover a more generous spirit in those who are liable to other imperfections, to draw a veil over their *real* or *imaginary* mistakes, and let them be buried in one common grave with their authors.

Mr. Ring insinuates, that I classed "New Theories in Medicine" with the Vaccine Practice, as if incapable of distinguishing between theory and practice; although so plain an allusion was made to the late doctrine advanced, respecting Burns and Scalds, and gouty inflammation.— But I scarcely wonder at this mistake; for as soon as his *favourite* subject was only hinted at, his thoughts were so absorbed in it, as to overlook all that was mentioned before in the former part of the paper; otherwise, any reader, I think, without the acumen of Œdipus, the interpreter of the fabulous enigma of Sphinx, might have easily understood it.

I am

I am further represented, indirectly, (if I mistake not) amongst other "obscure practitioners," as being only "a "pretended friend" to the Vaccine Interest. But where does Mr. R. gather that from? Is it because I believed it to be a *general,* though perhaps not an *universal* defence against variolous infection? Has not the Royal College of Physicians, in their candid and judicious Report, advanced the same sentiment? Since, therefore, I am found in such honourable company, the opinion of an individual, however popular in this interesting cause, whether I am only a "pretended friend" or an avowed enemy to it, can affect me but little.

Another reflection is, that Mr. R. cannot think me a friend to improvement in the healing art, by wishing to suppress whatever tends to that end from the press. Here he very much mistakes the point. My partiality for the science in particular, and regard for the welfare of mankind in general, totally forbid such a disposition.* I am well aware, that the most valuable discoveries in the different branches of medicine, had to contend with much opposition at first, through the medium of the press, and laudable exertions have been made to overcome them; but, when reduced to regular and established practice, it should seem superfluous to maintain any longer contests on the subject.

I hope from this statement it will appear, that I am not so inimical to useful communications from the press, as Mr. R. wishes to represent me. What I have said respecting a repetition of the same controversial subjects in the Journal, was not my language *only,* but that of other Readers of your useful Miscellany. It is expected in Literature, that writers should furnish us with subjects new as well as old; and so combine the *utile* with the *dulce,* as the *crambè repetita* will always cloy. And it is presumed the extensive scale upon which some of your Correspondents practice (for they are not all "obscure practitioners) must afford them diversified matter of information, calculated to promote the improvement of medical science.

Since writing the above, which was intended for the last Number of your Journal, another Commentator has appeared,

---

* As well might one be said to be an enemy to wholesome food or generous wine, because he drops a hint respecting the intemperate use of either.

appeared, under the (probably fictitious) signature of P. O. who affects to be much at a loss to understand the meaning of my communication. But as any farther explanation may not, by *him*, be deemed "profound," he must rest satisfied with what has been said in my "Answer to Mr. Ring," where it is stated, *when* medical subjects may be considered as "controversial" or "practical."—And I am the more disposed to this, as he thinks proper to conceal his real name, and shoot his arrows in the dark; for I heartily approve the maxim of a celebrated author, who used to assert, when addressed by anonymous Correspondents, that "writers of no name, deserve no notice."

I am, &c.

*Piccadilly, Nov.* 10, 1807. H. DAVIES.

